# Ambulatory function in spinal muscular atrophy: Age-related patterns of progression

**DOI:** 10.1371/journal.pone.0199657

**Published:** 2018-06-26

**Authors:** Jacqueline Montes, Michael P. McDermott, Elizabeth Mirek, Elena S. Mazzone, Marion Main, Allan M. Glanzman, Tina Duong, Sally Dunaway Young, Rachel Salazar, Amy Pasternak, Richard Gee, Roberto De Sanctis, Giorgia Coratti, Nicola Forcina, Lavinia Fanelli, Danielle Ramsey, Evelin Milev, Matthew Civitello, Marika Pane, Maria Carmela Pera, Mariacristina Scoto, John W. Day, Gihan Tennekoon, Richard S. Finkel, Basil T. Darras, Francesco Muntoni, Darryl C. De Vivo, Eugenio Mercuri

**Affiliations:** 1 Departments of Neurology, Columbia University Medical Center, New York, NY, United States of America; 2 Rehabilitation and Regenerative Medicine, Columbia University Medical Center, New York, NY, United States of America; 3 Department of Neurology, University of Rochester, Rochester, NY, United States of America; 4 Department of Biostatistics and Computational Biology, University of Rochester, Rochester, NY, United States of America; 5 Department of Neurology, Boston Children’s Hospital, Harvard Medical School, Boston, MA, United States of America; 6 Department of Physical Therapy and Occupational Therapy Services, Boston Children’s Hospital, Boston, MA, United States of America; 7 Department of Paediatric Neurology and Nemo Clinical Centre, Catholic University, Rome, Italy; 8 Dubowitz Neuromuscular Centre, UCL Great Ormond Street Institute of Child Health, London, United Kingdom; 9 Department of Physical Therapy, The Children’s Hospital of Philadelphia, Philadelphia, PA, United States of America; 10 Departments of Neurology and Pediatrics, Stanford University School of Medicine, Stanford, CA, United States of America; 11 Nemours Children’s Hospital, Orlando, FL, United States of America; 12 Departments of Neurology, Pediatrics, The Children’s Hospital of Philadelphia, University of Pennsylvania, Philadelphia, PA, United States of America; Iowa State University, UNITED STATES

## Abstract

Individuals with spinal muscular atrophy (SMA) type 3 are able to walk but they have weakness, gait impairments and fatigue. Our primary study objective was to examine longitudinal changes in the six-minute walk test (6MWT) and to evaluate whether age and SMA type 3 subtype are associated with decline in ambulatory function. Data from three prospective natural history studies were used. Seventy-three participants who performed the 6MWT more than once, at least 6 months apart, were included; follow-up ranged from 0.5–9 years. Only data from patients who completed the 6MWT were included. The mean age of the participants was 13.5 years (range 2.6–49.1), with 52 having disease onset before age 3 years (type 3A). At baseline, type 3A participants walked a shorter distance on average (257.1 m) than type 3B participants (390.2 m) (difference = 133.1 m, 95% confidence interval [CI] 71.8–194.3, p < 0.001). Distance walked was weakly associated with age (r = 0.25, p = 0.04). Linear mixed effects models were used to estimate the mean annual rate of change. The overall mean rate of change was -7.8 m/year (95% CI -13.6 –-2.0, p = 0.009) and this did not differ by subtype (type 3A: -8.5 m/year, type 3B: -6.6 m/year, p = 0.78), but it did differ by age group (< 6: 9.8 m/year; 6–10: -7.9 m/year; 11–19: -20.8 m/year; ≥ 20: -9.7 m/year; p = 0.005). Our results showed an overall decline on the 6MWT over time, but different trajectories were observed depending on age. Young ambulant SMA patients gain function but in adolescence, patients lose function. Future clinical trials in ambulant SMA patients should consider in their design the different trajectories of ambulatory function over time, based on age.

## Introduction

Spinal muscular atrophy (SMA) is a genetically determined neuromuscular disorder that affects motor neurons and their associated motor units, causing muscle atrophy and weakness. The most common cause of the SMA phenotype is a homozygous deletion of the Survival Motor Neuron (*SMN1*) gene located on chromosome 5q13. [1]Individuals with the mildest phenotype, SMA type 3, are able to walk independently, [2] but their residual weakness causes gait impairments and fatigue, [3–5] and ultimately can result in loss of ambulation. [2]

In the chronic forms of SMA, which include types 2 and 3, gross motor function worsens slowly over time with minimal changes observed in one year [6] and small changes over 4 years. [7] However, while SMA is thought to be a largely stable disorder, recent studies have highlighted different rates of progression based on age and function. [8–10] In non-ambulant patients, gross motor function improves until about age 5 years, and then worsens until age 15 years, followed by a relatively stable plateau phase in late adolescence and adulthood. [8] While ambulant patients have a similar trajectory of progression to that of non-ambulant patients when assessed using functional scales, [8] it has been suggested that a more specific assessment may be necessary to identify changes in ambulatory function. [11]

Assessments of walking ability are clinically relevant in this population. The six-minute walk test (6MWT) is a valid and reliable functional outcome measure in SMA. [12] Longitudinal studies have demonstrated minimal changes in the 6MWT over a 12-month period in a small, mixed cohort of children and adults. [13] While the majority of individuals had minimal changes in this study, greater changes were seen in children between the ages of 6 and 16.

The purpose of this study was to examine longitudinal changes in the 6MWT beyond one year in a larger cohort of SMA patients and to explore if changes in ambulatory function are associated with age or SMA subtype.

## Materials and methods

Data from three on-going prospective natural history studies in the US, Italy and UK were used in this investigation. Patients of any age or SMA type diagnosed with 5q SMA disease were offered participation. Exclusion criteria included patients with symptom onset of SMA after age 19 years and presence of unstable medical conditions. To reduce selection bias, all patients seen in neuromuscular clinics who fulfilled eligibility criteria were enrolled. Participants were scheduled to be evaluated every 6 months for up to 9 years. Only ambulatory participants who performed the 6MWT were included in the data analysis for this report. Those ambulatory participants with clinical symptoms before age 3 years were termed Type 3A and the others with the onset of clinical symptoms after age 3 years were termed Type 3B. [14] All three network specific natural history studies had local ethical approvals in place permitting the pilot of functional scales (SMA REACH UK: National Research Ethics Committee (REC) London Bromley, Health Research Authority REC reference 13/LO/1748; PNCR USA Institutional Review Boards (IRB) and Numbers: Columbia University Medical Center Human Research Protection Office IRB reference AAAE8252, The Children’s Hospital of Philadelphia IRB reference 10–007816, Boston Children’s Hospital Office of Clinical Investigations IRB reference 05-02-028, Stanford University Research Compliance Office IRB reference 31140). The Italian ethical requirements for natural history studies mean that the Italian SMA Network was not provided with an IRB number. All participants had given their explicit written informed consent to participate in the network specific natural history studies.

Motor function assessments were performed at every visit and determined based on functional ability in these natural history studies. The Children’s Hospital of Philadelphia Infant Test of Neuromuscular Disorders was used to evaluate SMA type 1 infants and non-sitters [15] whereas the Hammersmith Functional Motor Scale Expanded was used for those with SMA types 2 and 3 [16]. For participants who were able to walk without support for at least 10 meters, the Six-Minute Walk Test (6MWT) was used to assess ambulatory function. Since the focus of this study is ambulatory function, only 6MWT data were included in the analysis. Other details about the broader natural history study and outcome measures used are described in previous reports. [6, 7]

### Six-minute walk test (6MWT)

The 6MWT measures the maximum distance a person can walk in 6 minutes over a 25-meter linear course. It has been shown to be a valid and reliable assessment of exercise capacity and functional walking ability in SMA patients. [12] Distance walked over the entire 6 minute time period, distance covered each minute, and the time to complete each 25-meter interval were recorded. Successful completion was determined by the evaluator if the participant walked along the course without support or assistance for 6 minutes. Standing rests were permitted as often as needed but sitting was not permitted. Data were collected according to previously published guidelines [3] and identically across the three networks. Training was performed independently in US and European networks. As part of the activity of each network, evaluators used a common procedure manual [3] and were trained at in-person meetings.

### Statistical methods

Mean values of the 6MWT distance walked at baseline were compared between SMA subtypes 3A and 3B and between male and female participants using t-tests. Pearson’s correlation coefficient was used to quantify the association between age and 6MWT distance walked at baseline. Linear mixed effects models were used to estimate the mean annual rate of change (slope) for the whole group as well as by SMA subtype and age group (< 6, 6–10, 11–19, ≥ 20 years). Age groups were chosen to classify younger, intermediate, adolescent, and adult participants and are similar to those used in previous reports. [8] The model for estimating the overall mean slope included year of follow-up (continuous) with random intercept and slope coefficients. To make inferences about mean slopes in subgroups, the above model was expanded by including appropriate main effect and interaction terms in the model (e.g., age group and the age group by year interaction). Pairwise comparisons of mean slopes among the four age groups used a Bonferroni-corrected significance level of 0.05/6 = 0.0083.

## Results

Seventy-three ambulatory participants (33 female) who performed the 6MWT successfully more than once, at least 6 months apart, were included; follow-up ranged from 0.5 to 9 years. The mean age of the participants was 13.5 years (range 2.6–49.1), with 52 (71%) having disease onset before age 3 years (type 3A) and 21 (29%) having disease onset after age 3 years (type 3B) (Table 1). Overall, there were slightly more male participants (54.8%); however, the milder phenotype (type 3B) was predominantly male (85.7%).

**Table 1:** Baseline clinical characteristics for all participants by age and SMA subtype.

Variable	Type 3A(n = 52)	Type 3B(n = 21)	Overall(n = 73)
**Age (years)**	7.9 (6.9)	27.3 (12.4)	13.5 (12.4)
**Male (%)**	42.3	85.7	54.8
**Study (%)**			
***PNCRN***	36.5	42.9	38.4
***Italian SMA Network***	23.1	57.1	32.9
***SMA REACH UK***	40.4	0	28.8
**6MWT Total distance (m)**	257.1 (107.3)	390.2 (144.0)	295.4 (132.6)

Values are mean (standard deviation) or % as indicated

PNCRN = Pediatric Neuromuscular Clinical Research Network

6MWT = Six-Minute Walk Test

At the initial visit, the 6MWT ranged between 38 and 604 meters (mean ± standard deviation 295 ± 133 m); type 3B participants walked a longer distance on average (390 ± 144 m) than type 3A participants (257 ± 107 m) (difference = 133 m, 95% CI 72–194, p < 0.001) and male participants walked a longer distance on average (325 ± 137 m) than female participants (259 ± 121 m) (difference = 66 m, 95% CI 5–126, p = 0.03). Total distance walked was weakly associated with age (r = 0.25, p = 0.04).

**Fig 1:**
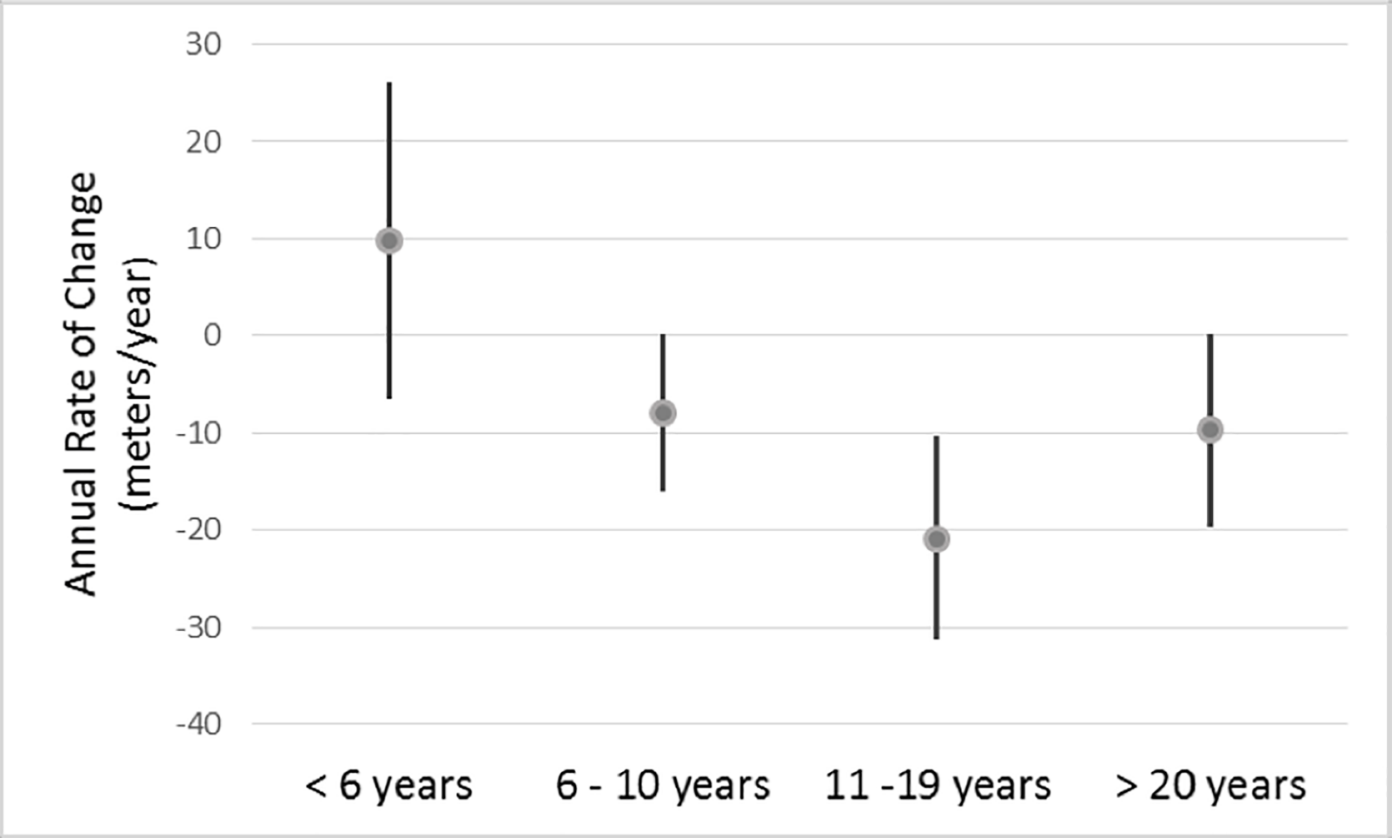
Mean annual rate of change in 6MWT distance and 95% confidence interval by age group.

**Fig 2:**
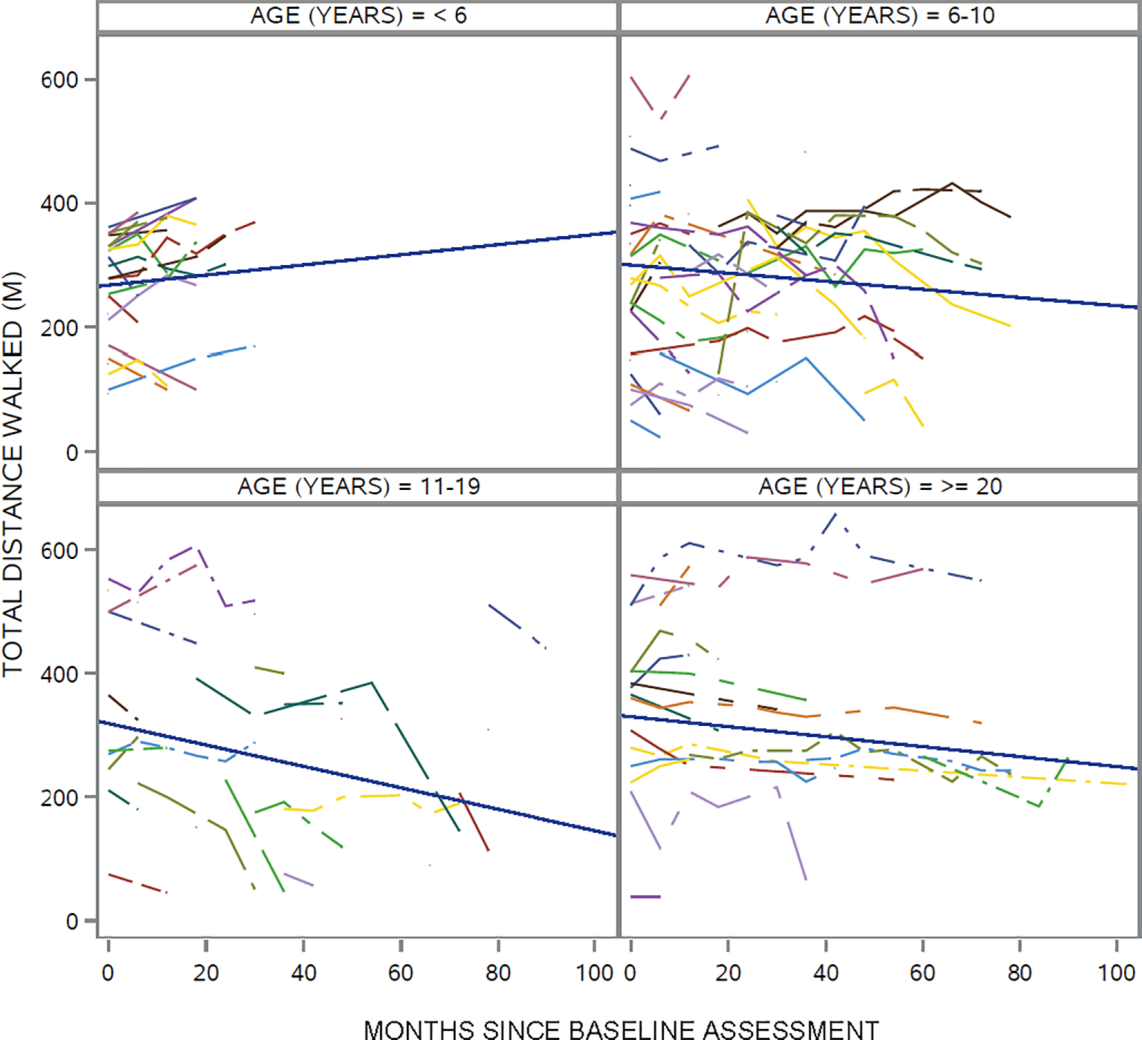
Individual trajectories and average trajectory of 6MWT distance by age group.

The mean rates of change in 6MWT distance, overall and by SMA subtype and age group, are provided in Table 2. The overall mean rate of change was -7.8 m/year (p = 0.009) and this did not differ by SMA subtype (type 3A: -8.5 m/year; type 3B: -6.6 m/year; p = 0.78) or gender (male: -10.0 m/year; female: -5.9 m/year; p = 0.51), but it did differ by age group (< 6: 9.8 m/year; 6–10: -7.9 m/year; 11–19: -20.8 m/year; ≥ 20: -9.7 m/year; p = 0.005 for the interaction between age group and time (Fig 1). The most prominent age group differences in mean annual rate of change were those between < 6 and 11–19 years (p = 0.0009) and between 6–10 and 11–19 years (p = 0.008) (Table 3). The individual trajectories by age group are shown in Fig 2.

**Table 2:** Mean annual rate of change on the 6MWT, overall and by subgroup.

	N	Mean Annual Rate of Change	95% CI	P-value
**Overall**	73	-7.8	(-13.6, -2.0)	0.009
**SMA subtype**				
***3A***	52	-8.5	(-15.2, -1.7)	0.02
***3B***	21	-6.6	(-17.7, 4.4)	0.24
**Age group**				
***< 6 years***	24	9.8	(-6.2, 25.9)	0.23
***6–10 years***	24	-7.9	(-15.7, -0.1)	0.05
***11–19 years***	10	-20.8	(-31.1, -10.6)	< 0.0001
***≥ 20 years***	15	-9.7	(-19.3, -0.1)	0.05

CI = Confidence interval

**Table 3:** Age group comparisons of mean annual rate of change on the 6MWT.

Comparison	Difference in Mean Annual Rate of Change	95% CI	P-value
**< 6 vs. 6–10 years**	-17.8	(-34.1, -1.4)	0.03
**< 6 vs. 11–19 years**	-30.7	(-48.6, -12.7)	0.0009
**< 6 vs. ≥ 20 years**	-19.6	(-38.0, -1.1)	0.04
**6–10 vs. 11–19 years**	-12.9	(-22.4, -3.4)	0.008
**6–10 vs. ≥ 20 years**	-1.8	(-13.8, 10.2)	0.77
**11–19 vs. ≥ 20 years**	11.1	(-2.5, 24.7)	0.11

CI = Confidence interval

## Discussion

Similar to non-ambulant patients, ambulant SMA individuals have different disease trajectories based on age. Ambulant SMA patients have improving walking ability until about age 6 years, a slowly declining phase until adolescence, then a steeper decline around puberty and until age 20 years, after which there is another slowly declining phase through adulthood. The age around puberty appeared to be the most vulnerable period in ambulant patients, as was also observed in our previous study using gross motor function scales. [8] Since hip and knee contractures and scoliosis are rarely a problem for ambulant SMA patients, weight gain and growth associated with puberty may contribute to progression in the setting of a limited neuromuscular system.

In this study, male participants walked farther than female participants on average. Our findings are in contrast to reports suggesting there may be genetic modifiers that are beneficial to female patients, particularly in the post-pubertal period. [17] These findings, however, should be interpreted with caution: while our overall study sample was largely balanced with respect to sex (56% male), nearly all of the type 3B patients (85.7%) were male. A greater representation of male patients in the milder cohort could have contributed to the overall sex differences in distance walked on the 6MWT. Future studies should explore if the distribution of sex differs according to phenotype among ambulatory SMA patients.

All clinical sites participating in this study are specialized neuromuscular centres in their respective geographical regions. The vast majority of patients referred are enrolled in the natural history studies. Rarely, does a patient or family refuse participation. While this should broadly represent the population at large, a selection bias secondary to the pattern of referrals to individual centres cannot be excluded. Importantly, the SMA 3 patient cohort included in this study is similar to those in previously reported natural history studies. [10, 14]

While patients with type 3B walked farther on the 6MWT on average at baseline, their mean rate of progression did not differ significantly from that of type 3A patients in this study. This is consistent with our previous observation using gross motor function scales where the rate of progression did not differ in ambulant subtypes over 1 year. [8] However, since the type 3A patients walked less overall, they may be at greater risk for losing ambulation.

On average, ambulant adolescent patients lost nearly 21 meters per year on the 6MWT in this study. While the precise impact of changes in the 6MWT on activities of daily living has not been explored in SMA, similar changes have been associated with decreased daily activities and participation in other neuromuscular disorders. [18] For clinical trials, stabilization of 6MWT distance in this population may therefore indicate an important therapeutic benefit.

None of the patients in our cohort lost ambulation. Further studies with longer follow up may help to prospectively capture loss of ambulation and to identify possible prognostic indicators of reaching this important endpoint. This would also help to identify if a number of factors such as changes in ankle range of motion, weight gain, progression of weakness or prolonged immobilization from injury may be considered as possible risk factors for loss of ambulation. This would permit proactive management and targeted intervention strategies.

While initially designed as an assessment of functional exercise capacity and responsiveness to treatment in cardiopulmonary disease, [19], the 6MWT has been incorporated in the functional evaluation of children with chronic pediatric conditions [20] and neuromuscular conditions including Duchenne muscular dystrophy [18] and SMA. [5, 13] Furthermore, the 6MWT has been included as a primary and secondary endpoint in clinical trials with children and adults with neuromuscular disease. [21–24]

With the availability of a disease modifying therapy [25] and additional emerging therapeutic interventions in development or clinical trials, [26] the potential for a new treated SMA phenotype is possible. [27] A need for objective and sensitive outcome measures targeting ambulatory, higher functioning children and adults can be anticipated. The 6MWT is a practical, easily administered assessment that is incorporated into clinical practice and management of SMA patients. Understanding the natural history of the disease and identifying disease trajectories as measured by the 6MWT is essential to interpret responsiveness to treatment in both a clinical and research settings.

## Conclusions

The 6MWT provides a global assessment of functional mobility, fatigue, strength and ability to walk. While overall walking ability slowly declined in the studied cohort over time, different trajectories were observed on the 6MWT in children and adults depending on age. Similar to non-ambulant SMA patients, young ambulant SMA patients gain function while in childhood, whereas early adulthood patients lose function. To date, treatment trials have mainly focused on infants and non-walkers. Future clinical trials will likely target the milder untreated ambulatory phenotypes. In these studies, the 6MWT should be considered as a possible important outcome measure. For study design, considerations for different trajectories of disease progression based on age (e.g., eligibility criteria, stratification) should be included.

## Supporting information

S1 Raw clinical data (XLSX)

## References

[1] Lefebvre S, Burglen L, Reboullet S, Clermont O, Burlet P, Viollet L, et al Identification and characterization of a spinal muscular atrophy-determining gene. Cell. 1995;80(1):155–65. . 7813012 10.1016/0092-8674(95)90460-3

[2] Darras BT. Spinal muscular atrophies. Pediatric clinics of North America. 2015;62(3):743–66. Epub 2015/05/30. doi: 10.1016/j.pcl.2015.03.010 . 26022173 10.1016/j.pcl.2015.03.010

[3] Montes J, Blumenschine M, Dunaway S, Alter AS, Engelstad K, Rao AK, et al Weakness and fatigue in diverse neuromuscular diseases. Journal of child neurology. 2013;28(10):1277–83. doi: 10.1177/0883073813493663 . 23847297 10.1177/0883073813493663

[4] Montes J, Dunaway S, Garber CE, Chiriboga CA, De Vivo DC, Rao AK. Leg muscle function and fatigue during walking in spinal muscular atrophy type 3. Muscle & nerve. 2014;50(1):34–9. doi: 10.1002/mus.24081 . 24122959 10.1002/mus.24081

[5] Montes J, McDermott MP, Martens WB, Dunaway S, Glanzman AM, Riley S, et al Six-Minute Walk Test demonstrates motor fatigue in spinal muscular atrophy. Neurology. 2010;74(10):833–8. doi: 10.1212/WNL.0b013e3181d3e308 . 20211907 10.1212/WNL.0b013e3181d3e308 PMC2839195

[6] Kaufmann P, McDermott MP, Darras BT, Finkel R, Kang P, Oskoui M, et al Observational study of spinal muscular atrophy type 2 and 3: functional outcomes over 1 year. Archives of neurology. 2011;68(6):779–86. doi: 10.1001/archneurol.2010.373 ; PubMed Central PMCID: PMC3839315. 21320981 10.1001/archneurol.2010.373 PMC3839315

[7] Kaufmann P, McDermott MP, Darras BT, Finkel RS, Sproule DM, Kang PB, et al Prospective cohort study of spinal muscular atrophy types 2 and 3. Neurology. 2012;79(18):1889–97. doi: 10.1212/WNL.0b013e318271f7e4 ; PubMed Central PMCID: PMC3525313. 23077013 10.1212/WNL.0b013e318271f7e4 PMC3525313

[8] Mercuri E, Finkel R, Montes J, Mazzone ES, Sormani MP, Main M, et al Patterns of disease progression in type 2 and 3 SMA: Implications for clinical trials. Neuromuscular disorders: NMD. 2016;26(2):126–31. doi: 10.1016/j.nmd.2015.10.006 ; PubMed Central PMCID: PMC4762230. 26776503 10.1016/j.nmd.2015.10.006 PMC4762230

[9] Iannaccone ST, Browne RH, Samaha FJ, Buncher CR. Prospective study of spinal muscular atrophy before age 6 years. DCN/SMA Group. Pediatric neurology. 1993;9(3):187–93. Epub 1993/05/01. . 8352849 10.1016/0887-8994(93)90082-n

[10] Wadman RI, Wijngaarde CA, Stam M, Bartels B, Otto LAM, Lemmink HH, et al Muscle strength and motor function throughout life in a cross-sectional cohort of 180 patients with spinal muscular atrophy types 1c-4. European journal of neurology. 2018;25(3):512–8. Epub 2017/12/02. doi: 10.1111/ene.13534 . 29194869 10.1111/ene.13534

[11] Finkel R, Bertini E, Muntoni F, Mercuri E. 209th ENMC International Workshop: Outcome Measures and Clinical Trial Readiness in Spinal Muscular Atrophy 7–9 November 2014, Heemskerk, The Netherlands. Neuromuscular disorders: NMD. 2015;25(7):593–602. Epub 2015/06/06. doi: 10.1016/j.nmd.2015.04.009 . 26045156 10.1016/j.nmd.2015.04.009

[12] Young SD, Montes J, Kramer SS, Marra J, Salazar R, Cruz R, et al Six-Minute Walk Test is Reliable and Valid in Spinal Muscular Atrophy. Muscle & nerve. 2016 doi: 10.1002/mus.25120 . 27015431 10.1002/mus.25120

[13] Mazzone E, Bianco F, Main M, van den Hauwe M, Ash M, de Vries R, et al Six minute walk test in type III spinal muscular atrophy: a 12month longitudinal study. Neuromuscular disorders: NMD. 2013;23(8):624–8. doi: 10.1016/j.nmd.2013.06.001 . 23809874 10.1016/j.nmd.2013.06.001

[14] Zerres K, Rudnik-Schoneborn S, Forrest E, Lusakowska A, Borkowska J, Hausmanowa-Petrusewicz I. A collaborative study on the natural history of childhood and juvenile onset proximal spinal muscular atrophy (type II and III SMA): 569 patients. Journal of the neurological sciences. 1997;146(1):67–72. Epub 1997/02/27. . 9077498 10.1016/s0022-510x(96)00284-5

[15] Glanzman AM, McDermott MP, Montes J, Martens WB, Flickinger J, Riley S, et al Validation of the Children's Hospital of Philadelphia Infant Test of Neuromuscular Disorders (CHOP INTEND). Pediatr Phys Ther. 2011;23(4):322–6. doi: 10.1097/PEP.0b013e3182351f04 . 22090068 10.1097/PEP.0b013e3182351f04

[16] O'Hagen JM, Glanzman AM, McDermott MP, Ryan PA, Flickinger J, Quigley J, et al An expanded version of the Hammersmith Functional Motor Scale for SMA II and III patients. Neuromuscular disorders: NMD. 2007;17(9–10):693–7. doi: 10.1016/j.nmd.2007.05.009 . 17658255 10.1016/j.nmd.2007.05.009

[17] Stratigopoulos G, Lanzano P, Deng L, Guo J, Kaufmann P, Darras B, et al Association of plastin 3 expression with disease severity in spinal muscular atrophy only in postpubertal females. Archives of neurology. 2010;67(10):1252–6. Epub 2010/10/13. doi: 10.1001/archneurol.2010.239 . 20937953 10.1001/archneurol.2010.239

[18] McDonald CM, Henricson EK, Abresch RT, Florence J, Eagle M, Gappmaier E, et al The 6-minute walk test and other clinical endpoints in duchenne muscular dystrophy: reliability, concurrent validity, and minimal clinically important differences from a multicenter study. Muscle & nerve. 2013;48(3):357–68. Epub 2013/05/16. doi: 10.1002/mus.23905 ; PubMed Central PMCID: PMCPMC3826053. 23674289 10.1002/mus.23905 PMC3826053

[19] ATS statement: guidelines for the six-minute walk test. American journal of respiratory and critical care medicine. 2002;166(1):111–7. Epub 2002/07/02. doi: 10.1164/ajrccm.166.1.at1102 . 10.1164/ajrccm.166.1.at1102 12091180

[20] Bartels B, de Groot JF, Terwee CB. The six-minute walk test in chronic pediatric conditions: a systematic review of measurement properties. Physical therapy. 2013;93(4):529–41. Epub 2012/11/20. doi: 10.2522/ptj.20120210 . 23162042 10.2522/ptj.20120210

[21] McDonald CM, Campbell C, Torricelli RE, Finkel RS, Flanigan KM, Goemans N, et al Ataluren in patients with nonsense mutation Duchenne muscular dystrophy (ACT DMD): a multicentre, randomised, double-blind, placebo-controlled, phase 3 trial. Lancet (London, England). 2017;390(10101):1489–98. Epub 2017/07/22. doi: 10.1016/s0140-6736(17)31611-2 . 28728956 10.1016/S0140-6736(17)31611-2

[22] Randeree L, Eslick GD. Eteplirsen for paediatric patients with Duchenne muscular dystrophy: A pooled-analysis. Journal of clinical neuroscience: official journal of the Neurosurgical Society of Australasia. 2018;49:1–6. Epub 2017/12/20. doi: 10.1016/j.jocn.2017.10.082 . 29254734 10.1016/j.jocn.2017.10.082

[23] Karaa A, Haas R, Goldstein A, Vockley J, Weaver WD, Cohen BH. Randomized dose-escalation trial of elamipretide in adults with primary mitochondrial myopathy. Neurology. 2018;90(14):e1212–e21. Epub 2018/03/04. doi: 10.1212/WNL.0000000000005255 ; PubMed Central PMCID: PMCPMC5890606. 29500292 10.1212/WNL.0000000000005255 PMC5890606

[24] Isayama R, Shiga K, Seo K, Azuma Y, Araki Y, Hamano A, et al Sixty six-month follow-up of muscle power and respiratory function in a case with adult-type Pompe disease treated with enzyme replacement therapy. Journal of clinical neuromuscular disease. 2014;15(4):152–6. Epub 2014/05/30. doi: 10.1097/CND.0000000000000029 . 24872213 10.1097/CND.0000000000000029

[25] Mercuri E, Darras BT, Chiriboga CA, Day JW, Campbell C, Connolly AM, et al Nusinersen versus Sham Control in Later-Onset Spinal Muscular Atrophy. The New England journal of medicine. 2018;378(7):625–35. Epub 2018/02/15. doi: 10.1056/NEJMoa1710504 . 29443664 10.1056/NEJMoa1710504

[26] Scoto M, Finkel RS, Mercuri E, Muntoni F. Therapeutic approaches for spinal muscular atrophy (SMA). Gene Ther. 2017;24(9):514–9. doi: 10.1038/gt.2017.45 . 28561813 10.1038/gt.2017.45

[27] Tizzano EF, Finkel RS. Spinal muscular atrophy: A changing phenotype beyond the clinical trials. Neuromuscular disorders: NMD. 2017;27(10):883–9. doi: 10.1016/j.nmd.2017.05.011 . 28757001 10.1016/j.nmd.2017.05.011

